# Buffering by Transporters Can Spare Geometric Hindrance in Controlling Glutamate Escape

**DOI:** 10.3389/fncel.2021.707813

**Published:** 2021-07-23

**Authors:** Leonid P. Savtchenko, Kaiyu Zheng, Dmitri A. Rusakov

**Affiliations:** UCL Queen Square Institute of Neurology, University College London, London, United Kingdom

**Keywords:** excitatory synapse, glutamate, glutamate spillover, glutamate transporters, astrocyte, perisynaptic astroglial processes, synaptic environment

## Abstract

The surface of astrocyte processes that often surround excitatory synapses is packed with high-affinity glutamate transporters, largely preventing extrasynaptic glutamate escape. The shape and prevalence of perisynaptic astroglia vary among brain regions, in some cases providing a complete isolation of synaptic connections from the surrounding tissue. The perception has been that the geometry of perisynaptic environment is therefore essential to preventing extrasynaptic glutamate escape. To understand to what degree this notion holds, we modelled brain neuropil as a space filled with a scatter of randomly sized, overlapping spheres representing randomly shaped cellular elements and intercellular lumen. Simulating release and diffusion of glutamate molecules inside the interstitial gaps in this medium showed that high-affinity transporters would efficiently constrain extrasynaptic spread of glutamate even when diffusion passages are relatively open. We thus estimate that, in the hippocampal or cerebellar neuropil, the bulk of glutamate released by a synaptic vesicle is rapidly bound by transporters (or high-affinity target receptors) mainly in close proximity of the synaptic cleft, whether or not certain physiological or pathological events change local tissue geometry.

## Introduction

Glutamatergic circuitry of the brain has long been associated with a “wired,” one-to-one type of transmission that carries excitatory signals between individual nerve cells. This type of connectivity has provided a basis upon which the computation logic of neural-network learning algorithms was established and benefited from. To ensure that glutamate released into the synaptic cleft does not escape activating its receptors beyond the target cell, excitatory synapses are often surrounded by perisynaptic astrocyte processes (PAPs), part of the sponge-like morphology of brain astroglia. PAPs vary extensively in shape and size, and their membrane surface is densely packed with high-affinity glutamate transporters, among other signalling molecules (reviewed in Heller and Rusakov, 2015; Bazargani and Attwell, 2016; Murphy-Royal et al., 2017; Rose et al., 2017; Verkhratsky and Nedergaard, 2018). At some specialised synaptic connections, PAPs form a comprehensive isolating shield around one or several synaptic contacts (Barbour, 1993; Grosche et al., 1999; Rollenhagen et al., 2007; Borst and Soria van Hoeve, 2012). However, common (small) cortical synapses normally have only a varied fraction of their immediate neuropil environment (20–80%) occupied by PAPs (Spacek and Harris, 1998; Ventura and Harris, 1999; Witcher et al., 2007; Lushnikova et al., 2009; Patrushev et al., 2013; Bernardinelli et al., 2014; Medvedev et al., 2014; Pannasch et al., 2014; Gavrilov et al., 2018; Henneberger et al., 2020). It has often been argued that the synapse has to be comprehensively surrounded by the transporter-enriched PAPs, to prevent synaptically released glutamate from spilling over to the neighbouring tissue. Whilst the latter would indeed stop glutamate from escaping, whether the commonly observed partial PAP coverage is as effective in this respect has remained uncertain, prompting intense theoretical and experimental exploration of extrasynaptic glutamate escape (Diamond, 2001; Rusakov, 2001; Scimemi et al., 2004; Szapiro and Barbour, 2007; Zheng et al., 2008; Scimemi et al., 2009; Henneberger et al., 2020).

This issue has been somewhat blurred by the “aqueous” connotation arising from the commonly used term “glutamate spillover.” In reality, glutamate molecules do not flow or spill over as do liquids. They undergo rapid Brownian diffusion, bouncing off multiple nanoscale obstacles (such as water molecules) millions of times, moving into random directions, in nanoscale steps. Thus, any diffusing glutamate molecule has a chance to encounter a PAP surface populated with high-affinity glutamate transporters. The other issue affecting our perception of extrasynaptic glutamate actions is our understanding of the extracellular space architecture. The classical electron micrographs of fixed brain tissue tend to depict the interstitial space as a system of thin gaps between adjacent cell membranes. It has emerged, however, that in live brain the extracellular space occupies ∼20% of neuropil tissue volume, with interstitial gaps sometimes as wide as 200 nm (Thorne and Nicholson, 2006; Tonnesen et al., 2018; Paviolo et al., 2020). These data suggest that there could be much less geometric hindrance to diffusion in the brain neuropil than commonly perceived. Our aim was therefore to understand better, in comparative terms, the roles of geometric hindrance and of glutamate transporter binding in regulating extrasynaptic escape of glutamate, as predicted by physics. To this end, we explored detailed Monte Carlo simulations of particle diffusion and (transporter) binding in complex, quasi-randomly shaped geometries representing the extracellular space.

## Methods

### Monte Carlo Simulations of Particle Diffusion

Monte Carlo algorithms for particle diffusion were designed and run with MATLAB: they were previously described in detail, and tested and constrained using various experimental settings (Savtchenko et al., 2013, 2021; Sylantyev et al., 2013). The simulation arena was a 3 μm wide cube, with 2,000 particles “released” instantaneously at the centre. Particles positioned at time *t* at point *r*_i_(*x*,*y*,*z*) were moved, over time step Δ*t*, to point *r*_*i* + 1_(*x* + 2δ_*x*_Δ_1D_,*y* + 2δ_*y*_Δ_1D_,*z* + 2δ_*z*_Δ_1D_) where Δ_1D_ stands for the mean square displacement in the Einstein’s diffusion equation for 1D Brownian motion $\Delta_{1\,D}^{2}=2\,D\,\Delta \,t$, *D* = 0.65 μm^2^/ms is the glutamate diffusion coefficient in the interstitial space (Zheng et al., 2017), and *δ_*x| y| z*_* denotes a “delta-correlated” (independently seeded, uncorrelated) uniform random number from the (–1, 1) range. The latter ensures that Brownian particles are equally likely to move into either direction whereas scale factor 2 for δ gives the average elementary displacement in *x-y-z* either –Δ_1D_ or +Δ_1D_. This algorithm provided the duty-cycle translational particle movements in a contiguous 3D space, over all directions with varied 3D steps, rather than over the rectangular 3D-lattice vertices used by us and many others previously. The randomness of the displacement vector helped avoid occasional numerical deadlocks for particles trapped near the space dead-ends formed by aggregated overlapped spheres. The time step Δ*t* (usually < 0.1 μs) was set to be small enough to prevent particles from “tunnelling” through the smallest obstacles, and the actual value of *D* was verified at regular intervals.

The interaction with obstacles was simulated either as an elastic collision, or as a permanent bond (the catchment layer of ± 3 nm of the sphere surface, comparable with the maximal elementary displacement Δ_1D_), with the probability as indicated. Because the characteristic diffusion time from the centre to the arena boundary (<1 ms) was much shorter than the time constant of glutamate unbinding from glial glutamate transporter (GLT1 type), particle binding to the spheres on the millisecond scale was set as permanent.

### Simulating Sphere-Filled Space Representing Brain Neuropil

There were at least two reasons to believe that randomly sized overlapping spheres would be a more realistic representation of neuropil compared to regular lattices of regular shapes, a tissue model used extensively by us and others previously. Firstly, multiple intersecting spheres give randomly shaped and randomly sized cellular elements and extracellular channels, as opposed to uniform or regular structures. Secondly, this approach provides a mixture of concave and convex shapes, including “diffusion dead-ends” which are considered an important trait of brain neuropil (Hrabe et al., 2004). Both features therefore reflect reality better than do regular lattices.

Filling the space with overlapping spheres followed the routines described in detail previously (Savtchenko et al., 2021). In brief, the key parameter controlling this procedure was the volume fraction β occupied by the spheres: β = 1-α where α commonly stands for medium porosity, such as the volume fraction of the extracellular space in brain tissue. The β-value was calculated by (a) scattering 10^5^ test points uniformly randomly throughout the arena, and (b) calculating the proportion of the point falling outside the spheres. We verified that increasing the number of such test points to 10^6^ altered β by < 1%, pointing to asymptotic accuracy.

To fill the space with overlapping spheres that have a distributed size, we generated random co-ordinates of sphere centroids across the arena, and the random radius value for each sphere, in accord with the designated diameter distribution, which in our case was uniformly random between 20 and 100 nm. The initial number of spheres was estimated based on their average volume and the average size (to give the required β-value), and we left the co-ordinate origin unoccupied by any sphere. The space-filling cycle was repeated, with adjusted sphere numbers, until β approached the required value with ∼5% accuracy.

Our initial tests revealed that introducing transporter binding effectively restricted free particle movement across the simulated arena within ∼1 ms. We therefore limited simulated time to 1 ms.

### Computing Environment

Monte Carlo simulations were run on a dedicated 8-node BEOWULF-style diskless PC cluster running under the Gentoo LINUX operating system (kernel 4.12.12), which was an upgraded, *ad hoc* built version of the cluster described earlier (Zheng et al., 2008). Individual nodes comprised an HP ProLiant DL120 G6 Server containing a quad-core Intel Xeon X3430 processor and 8 GB of DDR3 RAM. Nodes were connected through a NetGear Gigabit Ethernet switch to a master computer that distributes programs and collects the results on its hard disk. Parallelisation and optimisation of the algorithms and program codes were implemented by AMC Bridge LLC (Waltham, MA).

## Results

### Surface Binding Is Efficient in Curtailing Particle Diffusion Even in Highly Porous Environment

In a porous medium, the diffusion transfer rate scales, at least to a first approximation, with medium porosity α (Tartakovsky and Dentz, 2019), which in the brain represents tissue volume fraction of the extracellular space (Sykova and Nicholson, 2008). Thus, narrowing interstitial passages in the neuropil will slow down escape of glutamate released at the synapse. To understand how this would affect the scatter of glutamate molecules away from the release site, we simulated brain neuropil as a space filled by randomly sized, overlapping spheres representing cellular structures: this procedure formed a porous, randomly shaped medium, with volume fraction β occupied by spheres, or porosity α = 1-β (see section “Methods”) (Savtchenko et al., 2021).

**FIGURE 1:**
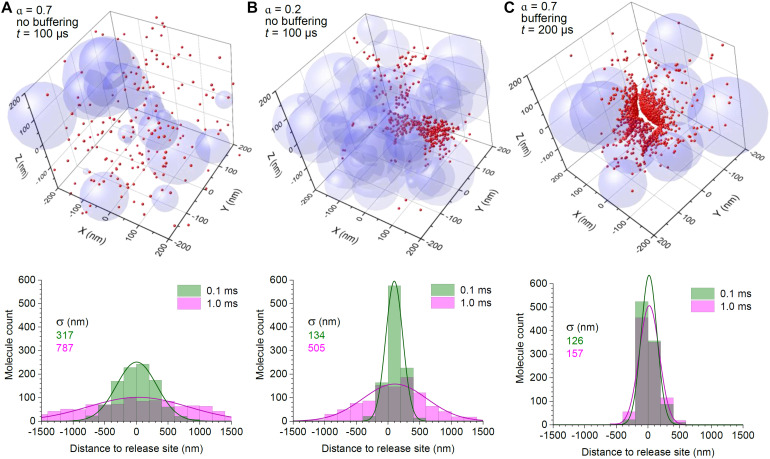
Surface binding to high-affinity transporters provides efficient control of extrasynaptic glutamate escape even in high-porosity tissue. **(A)** 3D graph: A snapshot of the simulated molecular scatter (400 nm wide fragment of the 3 μm wide simulation arena), 0.1 ms after release of 2,000 particles in the centre, with a scatter of overlapping spheres representing 3D obstacles to diffusion; α = 0.7, medium porosity (volume fraction of the free space). Histogram: spatial distribution of diffusing particles across the simulation arena, at two time points post-release, as indicated; solid lines, best-fit Gaussian distribution; σ, distribution dispersion (standard deviation). **(B)** Simulations as in **(A)**, but with medium porosity α = 0.2; note that the particle scatter is skewed because of the asymmetric geometry of diffusion obstacles (sphere aggregates); other notations as in **(A)**. **(C)** Simulations as in **(A)**, but with the particle-surface binding enabled; particle catchment occurs at a distance of ± 3 nm of the surface, to reflect the elementary diffusion displacement Δ_1D_ (see section “Methods”); other notations as in **(A)**.

For the sake of comparison, we first tracked the fate of 2,000 diffusing particles representing glutamate molecules released in the middle of a 3 μm cube arena, with space porosity of either α = 0.7 or α = 0.2, the latter representing an adult mammalian brain (Thorne and Nicholson, 2006; Tonnesen et al., 2018). The diffusion coefficient for glutamate was set at *D* = 0.65 μm^2^/ms, as measured through diffusion retardation in the interstitial brain space using time-resolved fluorescence anisotropy imaging (Suhling et al., 2015; Zheng et al., 2017). Because the characteristic free-diffusion time over the arena was < 1 ms, our simulations ran for 1 ms. The outcome showed that narrowing diffusion passages by 3.5 times slowed down particle escape, so that the molecular scatter became 1.5–2 times narrower (Figures 1A,B).

Next, instead of narrowing the extracellular space, we equipped surfaces of the spheres with the ability to hold diffusing molecules that “bumped” into them. This arrangement reflects the scenario when astroglial surfaces that are densely populated with GLT1 transporters (up to 10^5^ μm^–2^; Lehre and Danbolt, 1998; Lehre and Rusakov, 2002) represent all cell membranes in the nearby neuropil. Again, the time constant characterising glutamate unbinding or the glutamate uptake rate for the main glial glutamate transporter GLT1 is much longer than the diffusion time of < 1 ms (Bergles et al., 2002; Savtchenko et al., 2018). Thus, a permanent bond was fully representative of glutamate-transporter interaction on this time scale. Simulations showed that in these conditions the molecules remained within the vicinity of the release site, with little progression of the spread, even though 80% of the medium was available for free diffusion (Figure 1C).

### Glutamate Escape in Realistic Neuropil

The results above illustrate that, in principle, binding to glutamate transporters could provide an efficient barrier to diffusion even when the diffusion passages are widely open. However, it was important to relate these observations to a set of parameters characteristic of the real brain neuropil. Whilst α = 0.2 is thought to faithfully represent brain tissue porosity across regions (Nicholson and Phillips, 1981; Sykova and Nicholson, 2008; Tonnesen et al., 2018), astroglial coverage of synapse varies significantly. Stereological estimates based on quantitative electron microscopy suggest that in the neuropil of the rodent cerebellum (molecular layer) and hippocampus (area CA1), astroglial surfaces represent a ∼30 and 13% fraction, respectively, of all cell membrane surfaces (Lehre and Danbolt, 1998; Lehre and Rusakov, 2002; Savtchenko et al., 2018), whereas in the supraoptic nucleus cortex this fraction could exceed 50% (Pilgrim et al., 1982).

Based on these measurements, we first simulated glutamate release and diffusion in a modelled neuropil with α = 0.2 as in Figure 1B, but with the probability for individual molecules to be bound by the surface of either 0.3 or 0.13, thus representing the occurrence of transporter-enriched astroglial membranes in the cerebellar molecular layer or hippocampal area CA1, respectively. This approach assumes that the occurrence of astroglial and non-astroglial membranes near excitatory synapses does not follow any regular pattern but is arbitrary, which appears in line with the quantitative analyses of synaptic environment (Lehre and Rusakov, 2002; Patrushev et al., 2013; Medvedev et al., 2014). The other important assumption here is that the numbers of glutamate transporters expressed in perisynaptic astroglial membranes are much higher than the numbers of released glutamate molecules, a relationship consistent with single-vesicle release (Lehre and Danbolt, 1998; Savtchenko et al., 2013). We have also introduced a 320 nm wide, 20 nm thick synaptic cleft (free of transporters) based on the typical dimensions of such clefts at CA3-CA1 synapses (Harris and Stevens, 1989; Harris et al., 1992), centred at the arena co-ordinate origin, coinciding with the glutamate release site.

**FIGURE 2:**
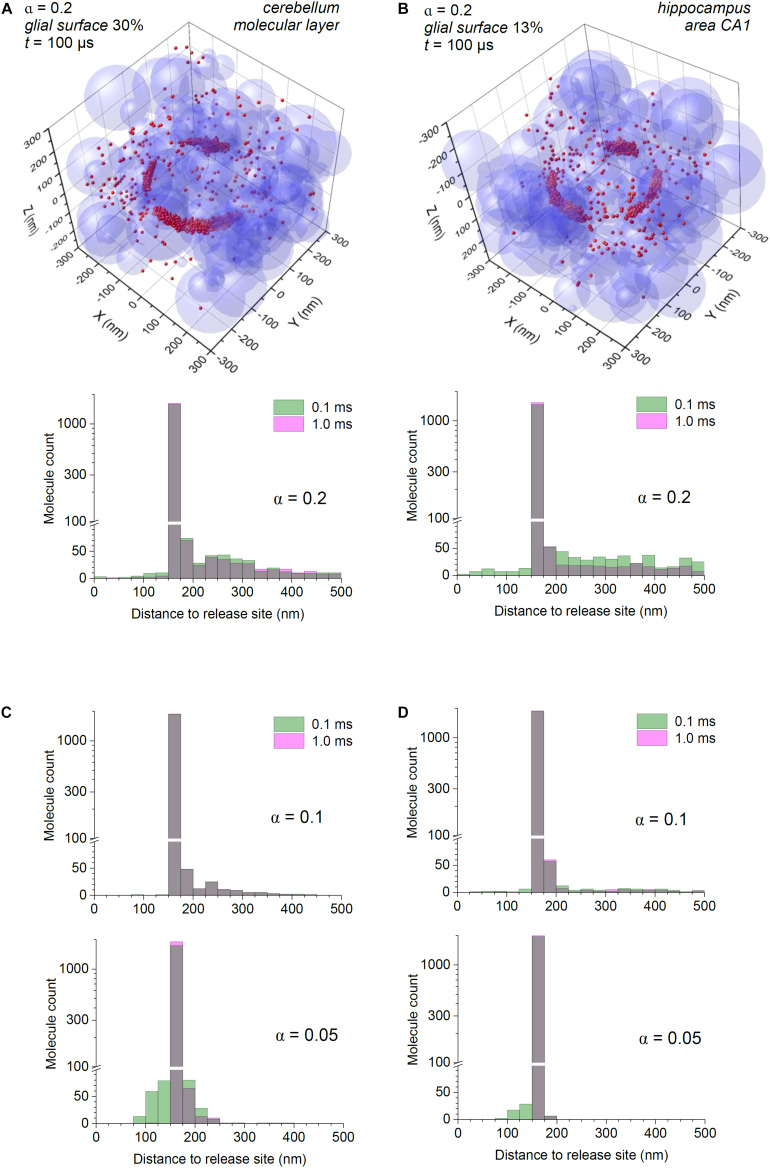
Astroglial high-affinity transporters control the scatter of escaping glutamate molecules in the modelled cerebellar and hippocampal neuropil. **(A)** Simulations as in Figure 1B, with the synaptic cleft (diameter 320 nm, height 20 nm) placed in the co-ordinate origin (also glutamate release site); particle-surface binding enabled, for each particle with probability of 0.3 (to mimic 30% chance of encountering cell membranes representing astroglia); medium porosity α = 0.2. Histogram: spatial distribution of glutamate molecules (free and transporter-bound combined) across the simulation arena, at two time points post-release, as indicated; note two scales (one log-scale) of the ordinate. **(B)** Simulations as in **(A)**, with the particle-surface binding enabled, for each particle with probability of 0.13 (to mimic 13% of neuropil cell membranes representing astroglia); other notations as in **(A)**. **(C)** Simulation outcome (molecule distribution histograms) for the model shown **(A)**, but with tissue porosity values α = 0.1 and α = 0.05, as indicated); other notations as in **(A)**. **(D)** Simulation outcome (molecule distribution histograms) for the model shown **(B)**, but with tissue porosity values α = 0.1 and α = 0.05, as indicated); other notations as in **(B)**.

The results suggest that the bulk of glutamate escaping from cerebellar molecular layer synapses is bound to transporters within ∼100 nm from the cleft, so that virtually all molecules become immobile very rapidly after ∼0.1 ms (Figure 2A). In these simulations, the distribution histograms represent all, both free and transporter-bound, molecules, so that the spatiotemporal dynamics of freely diffusing glutamate is reflected in how this distribution changes in time (see section “Discussion”). In the hippocampus, where astroglial presence is three times lower, the glutamate profile does change from 0.1 to 1 ms post-release, allowing for a more widespread “tail” of diffusing molecules, even though the majority of them still remain bound in close proximity to the cleft (Figure 2B).

### The Effect of Extracellular Space Shrinkage or Expansion

It has long been known that during intense excitatory activity, or in some pathological conditions such as epilepsy or ischemia, the extracellular space of the brain can shrink (Lux et al., 1986; Vorisek and Sykova, 1997; Vargova et al., 2001; Witcher et al., 2010). We have therefore asked whether such changes could significantly affect extrasynaptic escape of glutamate, by repeating our simulations for reduced porosity values. As expected, decreasing tissue porosity α from 0.2 to 0.1 and further to 0.05 led to a lower number of molecules escaping away from the cleft. However, the main feature of glutamate escape, its intense binding in cleft proximity, remained (Figures 2C,D).

**FIGURE 3:**
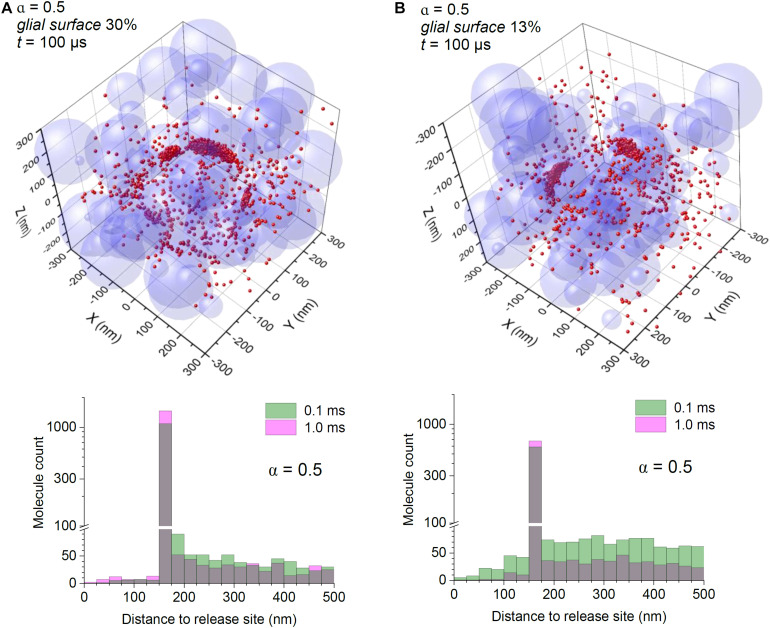
Astroglial high-affinity transporters control the scatter of escaping glutamate molecules under conditions of expanded extracellular space. **(A)** Simulations as in Figure 2, with the particle-surface binding enabled, for each particle with probability of 0.3 (to mimic 30% chance of encountering cell membranes representing astroglia); tissue porosity α = 0.5; other notations as in Figure 2A. **(B)** Simulations as in **(A)**, but with the particle-surface binding enabled, for each particle with probability of 0.13 (to mimic 13% of neuropil cell membranes representing astroglia); other notations as in **(A)**.

Finally, we asked what could happen when the extracellular space is significantly expanded, which is thought to be the case during postnatal development (Lehmenkuhler et al., 1993), but also in the human cerebellum (Cragg, 1979). Simulations adopting α = 0.5 still indicated perisynaptic binding as a prevalent feature even though glutamate molecules have a significantly wider spread, and a longer free-diffusion span than in cases with lower α (Figure 3).

## Discussion

### Scope and Limitations

It this study we asked which aspects of the perisynaptic environment are prevalent in controlling glutamate escape from excitatory synapses. Our primary purpose was therefore to understand whether geometric hindrance by tissue elements, and high-affinity binding by glutamate transporters, play comparable roles, in this context. The goal was neither to firmly establish the “true” glutamate escape profile for a particular synaptic type nor to expand such claims to various synaptic types featuring varied morphologies. The modelling relied on several basic assumptions, such as (a) single-vesicle glutamate release hence non-saturation of local glutamate transporters, (b) random distribution of glial and non-glial cellular surfaces in the neuropil, with the ratio established purely by the probability of encountering one or the other surface, and (c) negligible binding inside the synaptic cleft (numbers of glutamate receptors much smaller than that of released glutamate molecules). Clearly, these assumptions impose certain interpretability limitations: repetitive synaptic discharges or highly asymmetric occurrence of perisynaptic astroglia may produce a somewhat different dynamic picture of glutamate escape.

### Empirical Relevance

Our estimates are generally consistent with the previous theoretical assessments that used alternative modelling approaches (Scimemi et al., 2004; Zheng et al., 2008; Scimemi et al., 2009; Zheng and Rusakov, 2015; Armbruster et al., 2020) predicting a very rapid fall of free glutamate concentration the cleft. However, the distributions of mainly bound molecules obtained here could be particularly relevant to the experimental measurements of extrasynaptic glutamate escape using optical glutamate sensors (Hires et al., 2008; Jensen et al., 2019; Armbruster et al., 2020; Henneberger et al., 2020). Because such sensors feature the glutamate binding rate on the same scale as do glutamate transporters, one could simply assume that in our simulations a proportion of the binding sites represents glutamate sensors. In this case, the local sensor/transporter concentration ratio would reflect the ratio of glutamate molecules bound to the two respective targets. In other words, assuming no significant difference between the spatial arrangement of either transporters and sensors (e.g., as in the case of astroglia-expressing iGluSnFR), the distribution profiles obtained here for bound glutamate molecules should be equally relevant to sensor-bound molecules.

One may argue that the experimental fluorescence profiles of glutamate-bound iGluSnFR around synapses are much smoother, and with no “voids” indicating the synaptic cleft, compared with the profiles shown here (Jensen et al., 2019). However, this is most likely because fluorescence signal is blurred over the point-spread function of an optical system (∼0.4 μm in the *xy* plane and ∼1 μm in the *z* direction), and because imaged synapses will have their cleft randomly oriented with respect to the focal plane. Exploring 3D simulation results by mimicking optical projections to match a particular imaging setting should bring theoretical findings closer to a faithful representation of experimental readout.

In this context, one novelty element of the present simulations is tissue modelling that uses randomly sized, randomly positioned intersecting spheres. As explained in the section “Methods,” we believe that this approach should provide a fairer representation of brain neuropil than would regular lattices of regular shapes that we and others employed previously. Also, we presented examples of individual trials rather than averaged outcome because “average synapse geometry” is a non-existing entity in which important “outlier” features of perisynaptic architecture could be unduly smoothed out. We therefore considered it intuitively more revealing, in this particular case, to illustrate individual Monte Carlo realisations.

### Extracellular Space in Pathology

Our basic results suggest that glutamate transporters can efficiently restrict glutamate diffusion even when the diffusion escape passages are relatively open. As mentioned, dynamic changes of the extracellular space volume, such as its transient or long-term shrinkage, have long been associated with pathological brain conditions such as in epilepsy or stroke (Lux et al., 1986; Vorisek and Sykova, 1997; Sykova, 2001; Vargova et al., 2001; Witcher et al., 2010). At the same time, physiological studies of neurodegenerative diseases, stroke, or addiction have found reduced expression of glial glutamate transporters in brain tissue (Maragakis and Rothstein, 2004; Fontana, 2015; Kruyer et al., 2019), which normally undergo rapid recycling on astroglial surfaces (Michaluk et al., 2021). The present results suggests that, over a wide range of tissue porosities, high-affinity transporters remain the principal factor in curtailing glutamate escape. Thus, the availability of high-affinity glutamate transporters appears a prevalent mechanism to control extrasynaptic actions of glutamate in pathological conditions affecting brain tissue architectonics.

## Data Availability Statement

The original contributions presented in the study are included in the article/Supplementary Material, further inquiries can be directed to the corresponding author/s.

## Author Contributions

LS designed and carried out computer simulations. KZ designed and provided cluster computing facilities. DR narrated the study, designed specific tests, and wrote the manuscript. All authors contributed to the article and approved the submitted version.

## Conflict of Interest

The authors declare that the research was conducted in the absence of any commercial or financial relationships that could be construed as a potential conflict of interest.

## Publisher’s Note

All claims expressed in this article are solely those of the authors and do not necessarily represent those of their affiliated organizations, or those of the publisher, the editors and the reviewers. Any product that may be evaluated in this article, or claim that may be made by its manufacturer, is not guaranteed or endorsed by the publisher.
